# Primary triage in ED

**DOI:** 10.1186/1757-7241-21-S2-A8

**Published:** 2013-09-09

**Authors:** Vibeke Hald, Marianne Barylak

**Affiliations:** 1 Akutafdelingen Nykøbing F, 4800 Nykøbing F, Denmark

## Background

Clinical effectiveness and patient safety depends on standardization of the triage process. Through 4 years, nurses in our department have trained and used a 5-level national recommended triage model. A former study three years ago in our department showed variations in the triage evaluation between nurses with a kappa value at 0.45. Therefore, a new study was made to evaluate the accuracy of triage between nurses.

## Methods

Observational study of the triage praxis of 25 nurses, evaluated with audit of the electronic documentation of the triage process. Audit was performed using a set of explicit indicators every week with a sample of 20 patients in a 12 weeks period. The indicators were defined due to our standard protocol for triage. The following data were registered; name of the triage nurse, triage colour, and which observations the triage score was based on either vital signs, diagnosis or clinical evaluation. Data was cumulated and evaluated to identify if the nurses performed equally due to the standard.

## Results

Patients were mainly triaged due to vital signs 75%, 20% were triaged due to diagnosis and a smaller group 5% was triaged due to clinical evaluation. The main group of nurses had the same pattern of performance. Few nurses did only triage on the basis of diagnosis and forgot the vital signs, especially sepsis criteria.

## Conclusion

Triage determines to a large extend the resources committed to the patients in an ED. Therefore it is essential to know how equally the nurses perform triage. We observe that after three years of experience and training including classroom education, the majority of nurses use the standard principles of triage. Few nurses need greater insight and clarity, to distinguish between a given diagnosis and symptoms of serious illness.

